# Physical Activity and Bone: May the Force be with You

**DOI:** 10.3389/fendo.2014.00020

**Published:** 2014-03-03

**Authors:** Jonathan H. Tobias, Virginia Gould, Luke Brunton, Kevin Deere, Joern Rittweger, Matthijs Lipperts, Bernd Grimm

**Affiliations:** ^1^Musculoskeletal Research Unit, University of Bristol School of Clinical Sciences, Avon Orthopaedic Centre, Southmead Hospital, Bristol, UK; ^2^German Aerospace Center, Institute of Aerospace Medicine, Cologne, Germany; ^3^Atrium Medical Centre, AHORSE Foundation, Heerlen, Netherlands

**Keywords:** impact loading, bone, physical activity, BMD, exercise

## Abstract

Physical activity (PA) is thought to play an important role in preventing bone loss and osteoporosis in older people. However, the type of activity that is most effective in this regard remains unclear. Objectively measured PA using accelerometers is an accurate method for studying relationships between PA and bone and other outcomes. We recently used this approach in the Avon Longitudinal Study of Parents and Children (ALSPAC) to examine relationships between levels of vertical impacts associated with PA and hip bone mineral density (BMD). Interestingly, vertical impacts >4g, though rare, largely accounted for the relationship between habitual levels of PA and BMD in adolescents. However, in a subsequent pilot study where we used the same method to record PA levels in older people, no >4g impacts were observed. Therefore, to the extent that vertical impacts need to exceed a certain threshold in order to be bone protective, such a threshold is likely to be considerably lower in older people as compared with adolescents. Further studies aimed at identifying such a threshold in older people are planned, to provide a basis for selecting exercise regimes in older people which are most likely to be bone protective.

## Introduction

Physical Activity (PA) declines markedly in older people; less than 30% of 65- to 74-year-olds and less than 15% of adults >75 report any moderate-intensity PA lasting >10 min in the previous 4 weeks (1). As well as increasing physical frailty and co-morbidities, psychological, social, and economic factors contribute to this decrease. For example, the OPAL study, which used a socio-ecological approach to identify psycho-social and socio-environmental influences on PA as assessed by accelerometry (2), found relationships with the nature and frequency of outings (3), neighborhood social deprivation (4), lack of intrinsic motivation, and lack of an activity companion (5). Higher levels of PA benefit a wide range of physiological systems in older people, including cardiovascular, respiratory, metabolic, neurological and neuromuscular, and cognitive function^1^, and improve life expectancy (6). The WHO recommends that those above age 65 partake in a minimum of 150 min of moderate-intensity aerobic PA per week (e.g., brisk walking), or 75 min of vigorous-intensity PA (e.g., jogging)^2^.

## PA and Older People’s Bone Health

Hip fracture is a major cause of morbidity and mortality in older people, leading to loss of independence, and a huge economic burden through both direct medical costs and social sequelae (7). It is thought that age related declines in the intensity and quantity of PA contribute to this increase in risk of osteoporotic fracture, and that promotion of PA in older people helps to maintain bone mass: epidemiological studies report that risk of hip fracture is reduced in older adults who remain more physically active (8); walking for leisure is associated with reduced hip fracture risk (9–11). Therefore, although increased PA in the elderly leads to greater exposure to falls risk, it would seem that any tendency for this to increase fracture risk is outweighed by other benefits and that the net effect is a reduction in fracture risk. As well as benefits in terms of bone mass as described below, PA may also reduce the risk of falls through specific muscle-strengthening and balance-training activities, which preserve muscle strength, delaying sarcopenia, and maintaining neuromuscular function necessary to keep balance and react to a fall.

In terms of effects on bone mass, PA may stimulate bone formation and thus improve bone mineral density (BMD), which is strongly related to hip fracture risk (12), through exposing the skeleton to mechanical strain (defined as deformation of bone per unit length in response to loading). An important physiological link exists between exercise and bone, as demonstrated by findings from animal studies over 30 years ago that the skeleton is exquisitely responsive to mechanical strain; bone loss caused by immobilization was prevented by only four loading cycles per day (13). Though related to fracture risk, there is little evidence that walking interventions improve BMD, as judged by findings of a recent meta-analysis (14). In contrast, protocols that combined jogging, walking, and stair climbing consistently improve hip BMD in older people (15). Interventions to increase aerobic activities, high impact exercises, “odd-impact” exercise loading, and resistance training (designed to increase bone loading through increased muscle strength) also improve hip BMD in this group (15–19). However, the optimum type of activity for improving BMD remains unknown, and it is unclear whether a specific strain needs to be exceeded. Moreover, other aspects of impacts may also be important, such as movement frequency. In addition, specific activities may affect BMD at certain sites in preference to others, which may be important if improved BMD is to translate into reduced fracture risk which is the primary goal, in light of evidence that hip fracture risk is related to thinning of a specific portion of the femoral neck (20).

## Measurement of PA According to Level of Impact Load

Observational studies may be useful for estimating relationships between PA and bone outcomes, providing the PA measure in question is related to strain. PA questionnaires have been used to record participation in different sporting activities graded according to vertical impact loads (21). Pedometers were used in a cross sectional study of 105 individuals aged 49–64 years, with a dose–response relationship observed in females between cumulative loading as calculated from a combination of number of steps, walking speed, and weight, and hip BMD (22). Lower limb impact during weight bearing reflects their ground reaction force which is the product of mass times acceleration, and so depending on placement accelerometers can provide objective measures of exposure to different levels of impact load. To detect vertical movement of the center of mass, accelerometers need to be attached to the trunk, for example held in a belt laterally just below the waist, despite the fact that some dampening through the skin will occur particularly in obese individuals (other placements such as the ankle are less accurate as movements can occur independently of the center of mass). Using an Actigraph device in this way in adolescents from the Avon Longitudinal Study of Parents and Children (ALSPAC), vigorous PA (based on a threshold of 6200 cpm, equivalent to jogging) was positively related to cortical bone mass, but no independent relationship was seen for moderate PA after adjusting for vigorous PA (23).

Although these findings suggest that the Actigraph differentiates between PA exposure and bone outcomes according to impact level, earlier versions of this device were primarily designed to measure general body movement, and externally calibrated to energy consumption to be associated with obesity-related outcomes (24). They were limited in detecting brief high impact events with high osteogenic potential due to a narrow dynamic range (original devices had an upper range of 2.13g), filtering out of high frequency motion, and summation of records into epochs of typically 30–60 s. Moreover, rather than a true representation of “event” frequency, the counts per minute (cpm) output of the Actigraph integrates movement frequency with level of acceleration, making it difficult to relate the output to specific impacts or activities.

Newer generations of digital accelerometers [e.g., Actigraph GT3X-BT, Gulf Coast Data Concepts (GCDC) X16-1c] have wider dynamic ranges (8 and 16g, respectively), high sampling frequencies (>100 Hz), and the raw signal can be accessed without filtering or summation into epochs. Previous studies using a research prototype developed by Newtest suggest that the ability to derive impact loads from the raw signal, ideally suited for studying PA effects on the skeleton, can yield important insights. The Newtest prototype recorded the number of counts within 33 pre-specified acceleration bands, and distinguished exposure to high impact loads associated with osteogenic activities like running and jumping (25). In a prospective study of PA exposure in 64 premenopausal women using this device, a positive relationship was only observed between hip BMD and counts >3.9g (seen during running) (25). Similarly, after analyzing cross sectional relationships between exposure to different g-forces and bone development in ALSPAC 17-year-olds, hip BMD was most strongly related to counts >4g, in spite of their rarity, whereas no association was seen for lower impact loads after adjusting for exposure to higher impacts (26). This 4g threshold represents a higher impact than the 6200 cpm threshold for vigorous PA as used in our previous Actigraph study (23), but is entirely consistent with current understanding of skeletal physiology (27).

Subsequent analysis of pQCT-based measures performed at the mid-tibia suggested high impacts improve BMD of the lower limb through a combination of increased cortical thickness and periosteal circumference, with the latter effect strongest in boys (28). In future studies, we hope to repeat these measures to establish whether exposure to high impacts during childhood and adolescence has persisting effects on subsequent peak bone mass. Evidence that past history of sporting activity in childhood and adolescence is positively associated with cortical bone mass in young adult men is consistent with the suggestion that the positive influence of high impact activity on bone which we observed has a persisting effect (29).

Interestingly, impacts >3.1g (seen during jogging and running) were also particularly related to lean mass (30), suggesting this approach may also be more accurate in analyzing relationships with lean mass, with potential application to the study of sarcopenia. In contrast, impacts within 1–3g (e.g., moderately brisk walking and jogging (25) were most strongly related to fat mass (30). The latter relationship was equivalent to that previously reported from the same cohort based on moderate and vigorous physical activity (MVPA) as measured by Actigraph (31), consistent with cross calibration studies showing reasonably high correlation between Actigraph (MVPA), and the sum of counts in g bands >1.1g measured by Newtest (*r*^2^ = 0.41). Hence, exposure to lower impacts may be helpful in evaluating effects of PA on other systems.

## PA Impacts in Older People

While assessment of PA according to impact level has provided novel insights in adolescents and premenopausal women, it is unclear whether these findings also apply to older individuals. Even in adolescents, impacts >4g, or even >3.1g are rare (e.g., median 39 impacts >3.1g/day) (30). These impacts are likely to be even rarer in older people, but we are not aware of any previous studies examining this question. Therefore, we performed two pilot studies to characterize habitual exposure to PA in older people according to level of impact.

## Pilot Study Post Hip/Knee Replacement

We aimed to record habitual PA over 7 days in older people as part of a wider study of functional outcomes following hip/knee joint replacement surgery. We studied patients who were 3 months post joint replacement, by which time they had largely recovered from the effects of surgery and returned to their pre-operative functional level. After obtaining written informed consent, a GCDC Series X250-2 tri-axial accelerometer was attached to an elasticated belt, and worn in a horizontal orientation just anterior to the ischial crest during waking hours (except when washing, swimming, or bathing). Vertical impacts were classified into five bands (0.5–1, 1–2, 2–3, 3–4, >4g), and the mean count calculated at each band for each individual. Results were subsequently expressed as number of counts per day.

**Figure 1:**
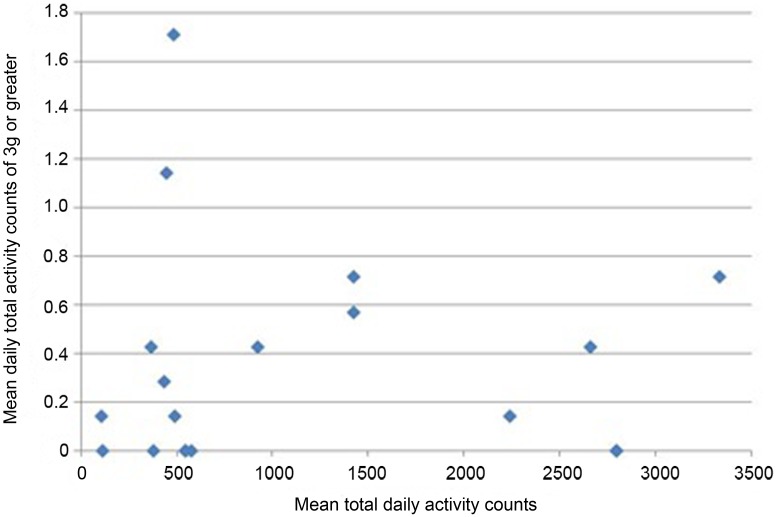
**Mean daily total activity counts plotted against mean daily vertical impacts >3g for each individual**.

**Figure 2:**
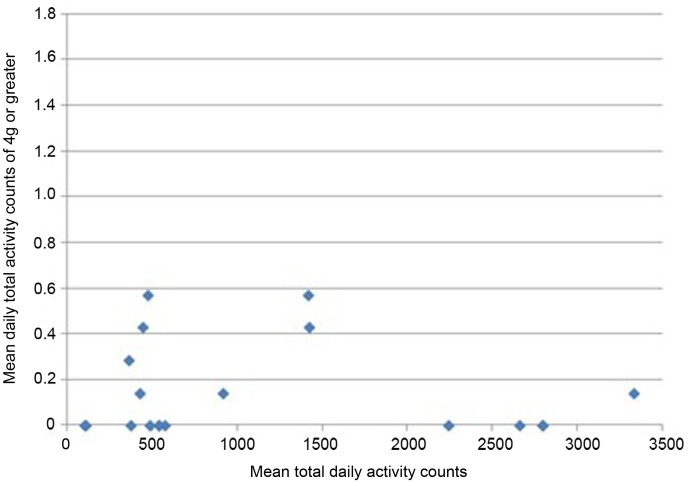
**Mean daily total activity counts plotted against mean daily vertical impacts >4g for each individual**.

All participants wore the monitors for the full 7 days (for a median of 106 h). Nineteen of 24 participants had usable data (median age 68.9). As a group, their level of function was relatively low as reflected by a median of 20 s for their 20 m walk time and 14 s for “get-up-and-go” test. Very few vertical impacts at the hip of 3g or higher were recorded in this study; 12 of the 19 participants achieved one or more impacts over 3g, with a maximum count of 8 impacts at this level over the 7 days (Table 1). Similarly, only 8 of the 19 achieved one or more impacts at the hip of 4g or greater, with a maximum of 4 impacts at this level recorded in the 7 days period. In order to investigate whether the most active individuals were also those achieving the highest vertical impacts, mean daily total counts against mean daily counts in the higher impact bands were plotted for each individual. The most active individuals, by total number of activity counts, were not necessarily those sustaining the highest vertical impacts (Figures 1 and 2).

**Table 1:** **Median and quartiles of the number of daily activity counts for each g band, and total activity counts, for 19 individuals 3 months post joint replacement**.

	Median	25th	75th
0.5–1g	512.86	373.71	1744.07
1–2g	72.86	29.00	89.36
2–3g	1.43	0.43	4.50
3–4g	0.14	0.00	0.21
>4g	0.00	0.00	0.21
Total activity count	538.57	434.29	1827.79

## Aerobics Class Pilot Study

Twenty participants were recruited from a group of older females regularly attending exercise classes at the University of Bristol Centre for Sports, Exercise and Health. Four study sessions, each with five participants, were held. Each participant was fitted with a tri-axial accelerometer as described above. Monitors were turned on just before the start of the session, and recordings matched to different activities within it. The study sessions consisted of a short exercise routine to music, similar to that carried out in the participants’ usual classes (Table 2). This was also extended by other activities (20 m walking normal pace, 20 m brisk walking, 20 cm step up and down with repeats, 30 cm step up and down with repeats). Data was analyzed as counts of vertical impacts for each activity, with impacts grouped in 0.3g bands (from 0–0.3 to >2.1g).

**Table 2:** **Aerobics class activities**.

1	“Mambo” leading from the left leg (left leg step forward, right leg step in place, left leg step backward).
2	“Mambo” leading from the right leg.
3	“Easy walk” leading from the left leg (Left foot forward, right foot forward and wide, left foot back, right foot next to left).
4	“Easy walk” leading from the right leg.
5	“Double side-step” (left foot sideways, right on spot, left foot next to right, right foot sideways, left on spot, right foot next to left).
6	“Half Jack” (jump to five-pointed star with arms to shoulder height, jump to standing with arms down, and feet together).
7	“Hamstring Curl” (alternate sides step sideways, bring other foot up to rear).
8	“Knee Lift” (lifting knee on alternate sides).

Participants were a mean of 67 years of age, and had a relatively good level of function as reflected by median of 14 s for 20 walk and 8 s for “get-up-and-go” test. For one participant, no data was recorded due to failure of the monitor. In another case, the monitor stopped working after the exercise class part of the session, but before the further individual activities. No vertical impacts higher than 2.1g were recorded in this study. One individual recorded 46 counts of 1.8–2.1g over all activities, and another 25 counts. Seven of the 19 participants with impact data achieved no counts in the 1.8–2.1g band, and in once case the highest impact recorded was in the 0.9–1.2g band.

## Future Research Questions

Taken together, these pilot studies suggest that not surprisingly, older individuals are exposed to considerably lower g-forces compared to adolescents and premenopausal women. For example, there was virtually a complete lack of higher impacts at the level suggested to be required for optimal bone development in adolescents. Due to the small size of the pilot studies presented here, and the selective nature of their recruitment, our findings are not necessarily generalizable to the wider population; in the Vertical Impacts and Bone in the Elderly (VIBE) study, we are in the process of extending our studies to characterize vertical impacts in much larger population-based cohorts of older people. Assuming our findings are at least partly representative of the level of vertical forces to which older people are exposed, impacts within lower g ranges which we recorded may well exert some protective effect on the skeleton. Loss of these low impacts may represent an important contribution to the development of osteoporosis in later life. The skeleton of older individuals may be more sensitive to low impacts compared to children and younger adults for several reasons. For example, lower g-forces may be needed to preserve bone, as opposed to stimulate its acquisition during peak bone mass attainment. In children and adolescents, bone accrual is achieved by a process of bone modeling involving a combination of longitudinal growth and periosteal expansion; it may well be that these physiological processes are regulated by a different level of strain, compared to bone remodeling responsible for preservation of bone in the mature skeleton. Furthermore, a given level of impact will produce greater strains in older people, due to their reduced bone strength.

Therefore, although a dose–response relationship between impact level and BMD may still exist in older people, this is likely to be shifted to the left. Defining such relationships will be key to identifying the types of activity that are likely to be the most effective in preventing bone loss and osteoporosis in older individuals. An important caveat is that exposure to such forces must be safe and without risk of injury. If forces between 1.8 and 2.1g, in the upper range of that observed in older participants performing an aerobics class, are found to be bone protective, it seems highly unlikely that these are sufficient to cause injury by themselves. However, performing such activities without supervision or appropriate training, or in the presence of co-morbidities affecting musculoskeletal or neurological function, may lead to a significant risk of falls and fractures. Therefore, having found which activities are likely to be bone protective, an important goal in their evaluation will be to ensure they can be delivered safely as well as effectively.

## Conflict of Interest Statement

The authors declare that the research was conducted in the absence of any commercial or financial relationships that could be construed as a potential conflict of interest.

## Footnotes

^1^http://www.health.gov/paguidelines/report/pdf/committeereport.pdf

^2^http://whqlibdoc.who.int/publications/2010/9789241599979_eng.pdf
